# A Polycomb-mir200 loop regulates clinical outcome in bladder cancer

**DOI:** 10.18632/oncotarget.5546

**Published:** 2015-10-17

**Authors:** Mónica Martínez-Fernández, Marta Dueñas, Andrew Feber, Cristina Segovia, Ramón García-Escudero, Carolina Rubio, Fernando F. López-Calderón, Claudio Díaz-García, Felipe Villacampa, José Duarte, María J. Gómez-Rodriguez, Daniel Castellano, José L. Rodriguez-Peralto, Federico de la Rosa, Stephan Beck, Jesús M. Paramio

**Affiliations:** ^1^ Molecular Oncology Unit, CIEMAT (ed70A), 28040 Madrid, Spain; ^2^ Universitary Hospital 12 de Octubre, Research Institute 12 de Octubre i+12, 28041 Madrid, Spain; ^3^ Medical Genomics, UCL Cancer Institute, University College London, London WC1E 6BT, UK; ^4^ Uro-oncology Section, Universitary Hospital 12 de Octubre, 28041 Madrid, Spain; ^5^ Anatomic Pathology Service, Universitary Hospital 12 de Octubre, Research Institute 12 de Octubre i+12, 28041 Madrid, Spain

**Keywords:** miRNA, Polycomb, bladder cancer, recurrence, epigenetics

## Abstract

Bladder cancer (BC) is a highly prevalent disease, ranking fifth in the most common cancers worldwide. Various miRNAs have recently emerged as potential prognostic biomarkers in cancer. The miR-200 family, which repressed the epithelial-to-mesenchymal transition (EMT), is repressed in multiple advanced cancers. However, its expression and function in BC is still poorly understood. Here we show that miR-200 family displays increased expression, probably due to the activation of specific oncogenic signaling pathways, and reduced promoter methylation, in BC compared to normal bladder samples. Furthermore, we show that the expression of these miRNAs is decreased in high grade and stage tumors, and the down-regulation is associated with patient's poor clinical outcome. Our data indicate that the miR-200 family plays distinct roles in Non-Muscle (NMIBC) and Muscle-Invasive BC (MIBC). In MIBC, miR-200 expression post transcriptionally regulates EMT-promoting transcription factors ZEB1 and ZEB2, whereas suppresses BMI1 expression in NMIBC. Interestingly, we show that increased EZH2 and/or BMI1 expression repress the expression of miR-200 family members. Collectively, these findings support a model of BC progression through a coordinated action between the Polycomb Repression Complex (PRC) members repressing the miR-200 expression, which ultimately favors invasive BC development. Since pharmacological inhibition of EZH2 in BC cell lines lead to increased miR-200 expression, our findings may support new therapeutic strategies for BC clinical management.

## INTRODUCTION

Bladder cancer (BC) is the fourth and fifth most commonly diagnosed male malignancy in the United States and Europe, respectively. The current BC clinical management is primarily dictated by the stage pathological characteristics. At diagnosis, approximately 70% of the patients present with superficial non muscle invasive BC (NMIBC; stages Ta/T1), which are usually treated by transurethral resection (RTU), followed by instillation with BCG or Mitomycin C in certain cases. The remaining 30% cases present with muscle invasive BC (MIBC; stages T2–T4) and are usually at a high risk of death associated with distant metastasis [1, 2]. The MIBC are usually treated by cystectomy and chemotherapy. Remarkably, there have been no significant improvements in BC therapies for the last decades. NMIBC management is also complicated because it displays high recurrence rates. A significant fraction of NMIBC recurs as MIBC, with the concomitant increased risk for the patients. As a consequence, NMIBC requires a systematic follow up by cystoscopy implying important morbidity problems and producing a prominent cost for health care systems [3, 4]. Despite originating from the urothelium, NMIBC and MIBC may differ at the molecular level [5–8]. However, the tumor progression in recurrences suggests that some NMIBC are early diagnosed MIBC (prior to the invasion of bladder muscle layer). This is supported by the recently identification of possible BC intrinsic subgroups on the basis of specific biomarkers [8]. Nonetheless, the development of potential biomarkers of NMIBC that may identify tumors at high risk of recurrence and progression is a current need. These may also help to determine new targeted therapies for BC management.

Epigenetics alterations are heritable but reversible modifications that modify gene expression without changing primary DNA sequences. Epigenome functions are fundamental for the normal status of gene expression and its alterations affect basic cellular processes such as proliferation, differentiation and apoptosis, which may lead to important diseases including cancer [9]. Therefore, epigenetic-based cancer biomarkers are promising tools for detection, diagnosis, assessment of prognosis, and prediction of response to therapy [10]. Epigenetic processes include DNA methylation, microRNA expression, chromatin remodeling, and histone modifications.

Among the histone modifiers, the polycomb repression complex (PRC) proteins are key elements. They act primarily as transcriptional repressors through histone modifications leading to chromatin remodeling and control the expression of multiple developmentally-regulated genes. PRCs have also been involved in cancer pathogenesis, in part through promoting an epithelial-mesenchymal transition (EMT) genetic program [11, 12]. In addition, the PRCs contribute to maintain pluripotency and self-renewal in embryonic and cancer stem cells [13–16]. PRCs may appear in two different biochemically and functionally classes, depending on their core protein components and their specific histone modifying activity: PRC1 includes the ubiquitin ligases BMI1 and RING1 and ubiquitinates H4; PRC2 contains EED, SUZ12 and EZH2 and generates H3K27me3 marks [15, 16]. The possible involvement of PRC in bladder cancer has been reported in close association with tumor pathogenesis, aggressiveness and the presence of putative cancer stem cells (CSCs) [17–20]. More recently we have shown that EZH2, the catalytic subunit of PRC2, governs a gene circuitry leading to increased recurrence and progression in human and in a transgenic mouse model of NMIBC [21].

The importance of miRNAs as epigenetic mechanism is also well-known, not only for normal development, but also for human disease, including cancer. Several studies have shown their role both as tumor suppressors and oncogenes [22, 23]. Moreover, there is critical crosstalk between various miRNAs and PRC [24–27]. However, such relationships are not well characterized in bladder cancer. Here we report the role of epigenetic deregulation of miRNA expression, DNA methylation and PRC activation plays in bladder cancer with distinct and differing roles in NMIBC and MIBC, providing a framework that might explain the tumor progression in recurrences of NMIBC.

## RESULTS

### miR 200 family is overexpressed in bladder tumor samples

**Figure 1:**
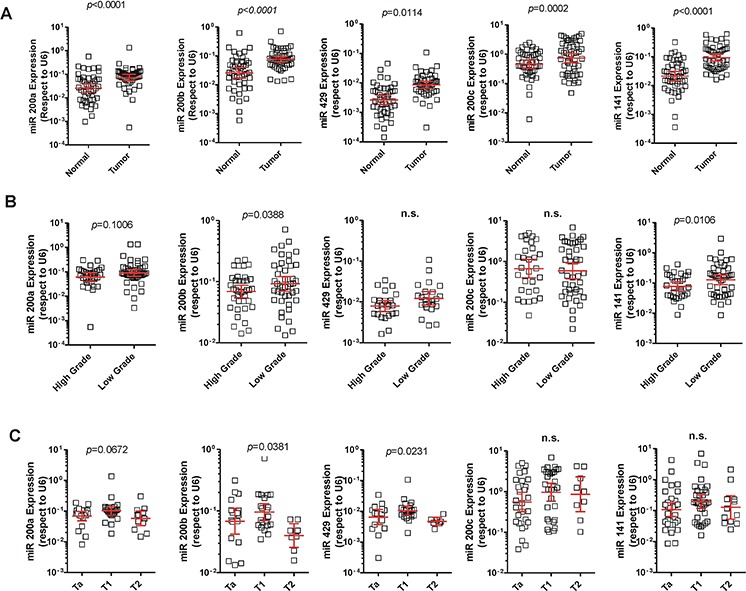
Expression of the miR-200 family in NMIBC **A.** qPCR analyses showing increased expression of the miR-200 family in NMIBC compared to normal samples. **B.** qPCR analyses showing increased expression of the miR-200 family in low grade compared to high grade tumors. **C.** qPCR analyses showing reduced expression of the miR-200 family in T2 tumors (muscle invasive) in comparison with Ta and T1 tumors (non-muscle invasive).

To identify potentially deregulated miRNA species in NMIBC samples, we compared the miRNA profiles of 28 NMIBC and 10 normal bladder samples from a recently reported microarray data [21]. This analysis revealed that all five members of the miR-200 family were significantly upregulated (Table 1). The miR-200 family includes 5 members located in two distinct genomic clusters: one, located on chromosome 1, encodes miR-200a, miR-200b, and miR-429, while another cluster, on chromosome 12, encodes miR-200c and miR-141. The microarray data revealed that miRNAs of both clusters were upregulated in tumors (Table 1). To validate this finding, we analyzed the expression of miR-200 family members by qPCR in a series of normal and NMIBC samples with known clinicopathological characteristics (Sup. Table 1). We found that all miR-200 family members displayed increased expression in tumors compared to normal samples (Fig. 1A). However, when tumors were classified according the grade, we observed lower expression in high grade respect to low grade tumors (Fig. 1B). Looking for differences among tumor stages, a similar trend was found for the whole family, with a moderate decrease in T2 stage samples, characterized by tumor invasion of the muscle layer (Fig. 1C).

**Table 1:** The top most up-regulated miRNAs in tumor vs normal bladder samples

Probe Set ID	Gene Symbol	mRNA Accession	Fold change (norm/tum)	*p* Value
8141419	MIR25	NR_029498	0,3355559	<10^−4^
8087250	MIR425	NR_029948	0,39621726	<10^−4^
8141423	MIR106B	NR_029831	0,34435254	<10^−4^
**7953590**	**MIR200C**	**NR_029779**	**0,41392165**	**<10^−4^**
8141421	MIR93	NR_029510	0,37645748	<10^−4^
**7896859**	**MIR200B**	**NR_029639**	**0,3764013**	**<10^−4^**
**7896861**	**MIR200A**	**NR_029834**	**0,45742458**	**<10^−4^**
7955906	MIR148B	NR_029894	0,5241288	<10^−4^
7957608	MIR492	NR_030171	0,5220465	<10^−4^
8083737	MIR15B	NR_029663	0,37378508	<10^−4^
8175250	MIR19B2	NR_029491	0,4328785	<10^−4^
**7953592**	**MIR141**	**NR_029682**	**0,51092947**	**<10^−4^**
8031037	MIR517C	NR_030214	0,51400614	<10^−4^
7969574	MIR622	NR_030754	0,43636504	<10^−4^
8067277	MIR296	NR_029844	0,5251195	<10^−4^
8142880	MIR182	NR_029614	0,46941015	<10^−4^
8073822	MIRLET7A3	NR_029478	0,38271952	<10^−4^
8049682	MIR149	NR_029702	0,6081797	<10^−4^
8127500	MIR30A	NR_029504	0,48555717	<10^−4^
7949275	MIR194–2	NR_029829	0,5730759	<10^−4^
8016400	MIR152	NR_029687	0,46595502	<10^−4^
8120206	MIR206	NR_029713	0,49778116	<10^−4^
8175248	MIR92A2	NR_029509	0,48623604	<10^−4^
7997008	MIR140	NR_029681	0,46796805	<10^−4^
8063921	MIR1–1	NR_029780	0,52695054	0,0123
7949273	MIR192	NR_029578	0,6647741	0,01013
8175252	MIR106A	NR_029523	0,49169168	0,01014
8149277	MIR124–1	NR_029668	0,4849764	0,010149
8146643	MIR124–2	NR_029669	0,55872	0,01014
**7896863**	**MIR429**	**NR_029957**	**0,6116756**	**0,01014**

Data came from Limma analyses of previously reported microarray results (GSE38264). The miRNA name, the logarithm fold change, and the *p*-value are represented for each case. The miR-200 family members are in bold.

**Figure 2:**
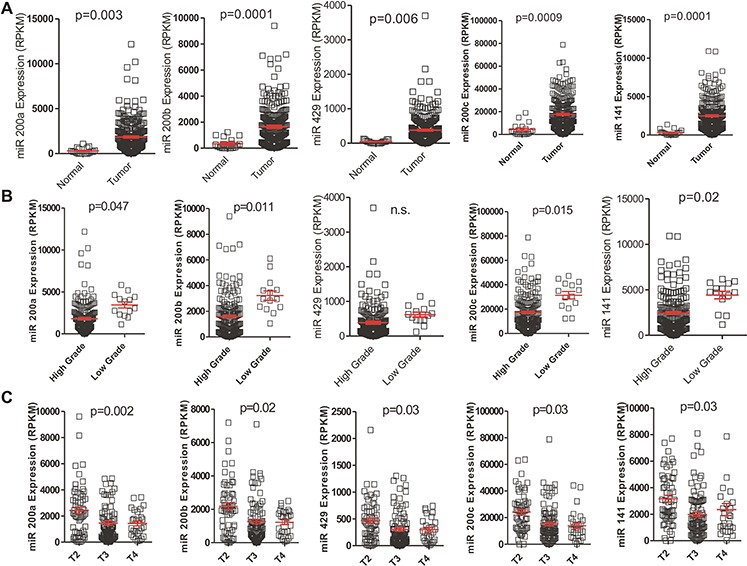
Expression of the miR-200 family in MIBC **A.** Increased expression of miR-200 family in tumor samples compared to normal samples present in the external TCGA dataset. **B.** Decreased expression of miR-200 family members in high grade MIBC present in the TCGA external dataset. **C.** Decreased miR-200 expression between T2 and T3 MIBC samples present in the external TCGA dataset.

To further substantiate our findings, we used the miRNA-seq data available at The Cancer Genome Atlas (TCGA) Database. This includes 251 MIBC and 19 normal bladder samples. We found up-regulation of miR-200 family members in tumors compared to normal bladder, which was particularly noticeable for cluster 2 (miR141 and miR-200c) miRNAs (Fig. 2A). In agreement with NMIBC, TCGA samples also showed higher expression in low grade tumors for all of the miR200s members (Fig. 2B). Regarding the tumor stage, a decreased expression was observed between T2 and T3 samples, when the tumor has grown through the muscle into the fat layer (Fig. 2C).

### Hypomethylation causes miR200 upregulation in BC

**Figure 3:**
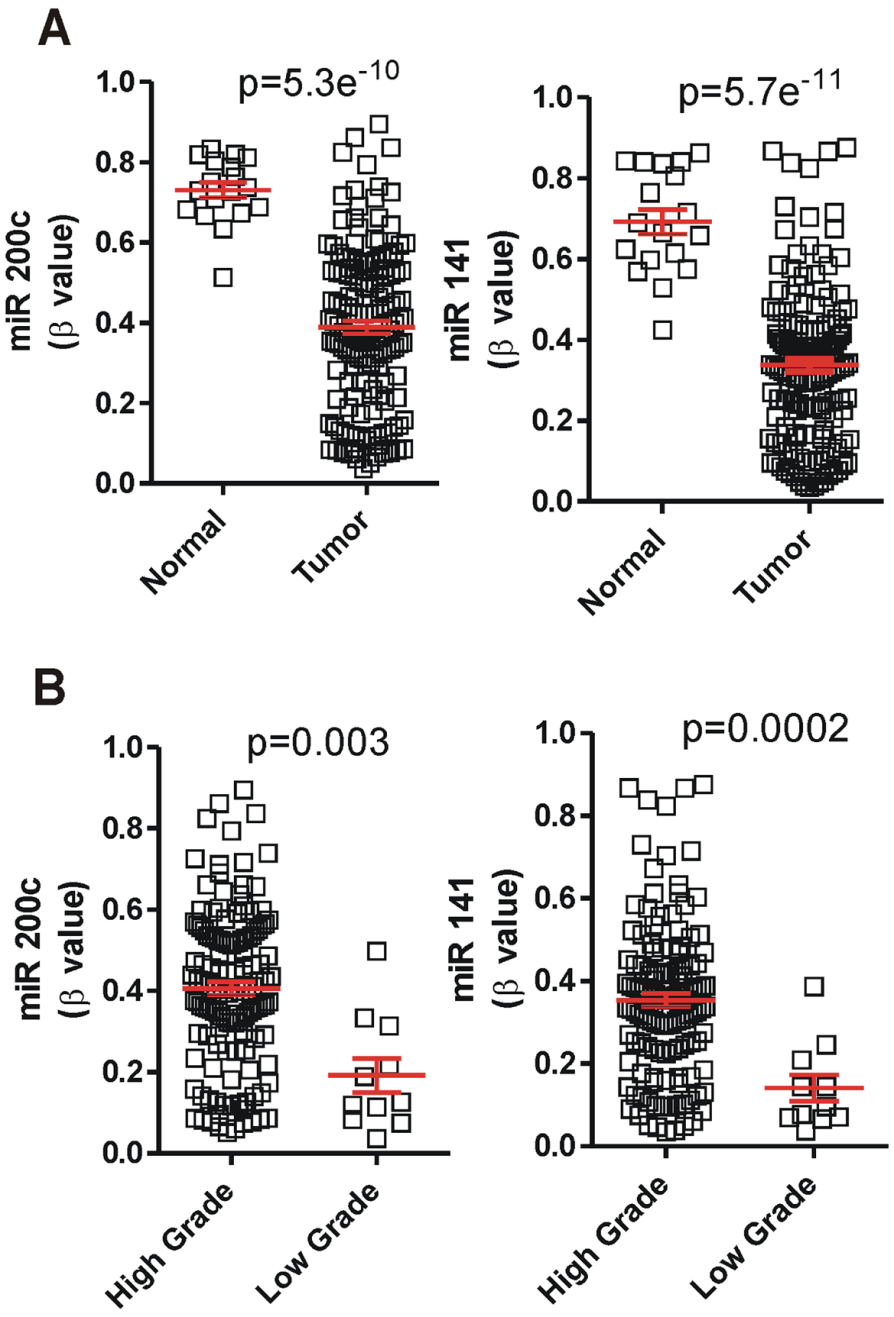
The expression of miR-200 is increased by hypomethylation in MIBC **A.** Tumors displayed miR-200 loci hypomethylation in comparison with normal samples present in the TCGA database. **B.** High grade tumors showed hypermethylation in cluster 2 when comparing with the low grade tumors present in TCGA data.

To obtain a possible mechanistic explanation of altered levels of miR-200 family members, we also analyzed the corresponding TCGA data to determine possible differences in gene methylation levels. We found that the family showed a significant decrease in methylation in the tumor samples (*p* values ≤ 10^−10^), this was highly significant in the cluster 2 of the miR-200 family (Fig. 3A). In addition, comparison of the methylation between different tumor grades showed increased methylation in the high-grade samples characterized by reduced miR-200 expression (Fig. 3B).

### Functional relevance of miR-200 upregulation in BC

**Figure 4:**
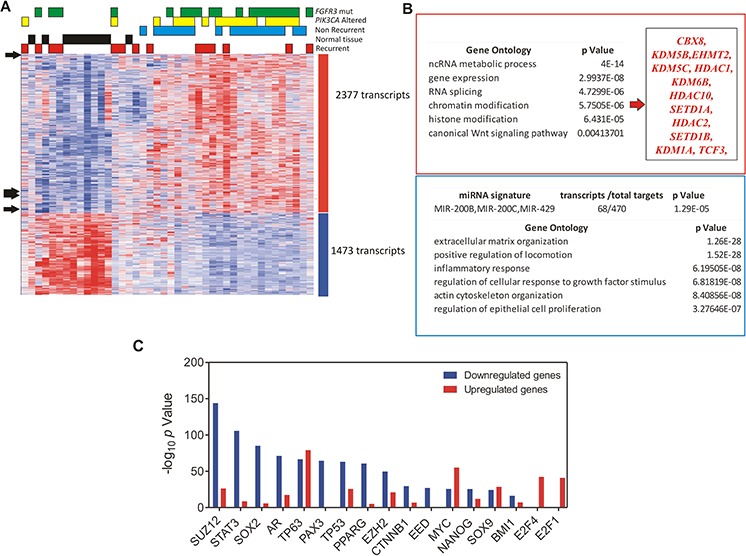
Analysis of genes displaying similar or opposite expression pattern respect to miR-200 family members **A.** Heatmap showing the unsupervised classification of genes (rows) according to the miR-200 family pattern, using the Plavidis Template Matching (PTM) approach in the TMEV utility (*p* ≤0.005). Black arrows show the position of miR200 members. Each column represents a sample. A red (overexpressed) to blue (downregulated) scheme following the above scale limits (in log2 scale) is shown. Numbers on the right denote the number of transcripts of each group (upregulated or downregulated). The unsupervised clusterization showed 2377 transcripts with a similar pattern to miR-200 members, and 1473 transcripts displaying opposite trend. **B.** Gene Ontology of Biological Processes of the upregulated (in the red box) and downregulated transcripts (in the blue box). **C.** Summary of Chip Enrichment Analysis showing the putative binding of transcription factors to genes displaying an expression pattern similar (red bars) or opposite (blue bars) to miR-200 family members.

To analyze the functional relevance of miR200 family upregulation, we classified our previous mRNA expression microarray data according to the miR200 family pattern (see Methods). This showed that 2377 transcripts followed a similar pattern to that of miR200s, whereas 1473 transcripts display opposite trend (Fig. 4A; Supplementary Tables 2 and 3). Among the genes displaying opposite trend, we found significant overlap with multiple targets of the miR-200 family, indicating that miR-200s increased expression might have functional relevance in BC pathogenesis (Fig. 4B). The unsupervised classification (Fig. 4A) also showed that tumors bearing *FGFR3* gene mutations and/or *PIK3CA* gene alterations (mutations or copy gains) usually clustered together, following the miR200 pattern. Nonetheless, when we compared miR-200 family member expression across the patient series, no significant differences were found according *FGFR3* and/or *PIK3CA* gene status (not shown), suggesting that these oncogenic alterations are not the main responsible for such increased expression.

Gene Ontology analysis showed that those genes displaying an inverse correlation with the miR-200 expression pattern were primarily involved in extracellular matrix organization, cell migration, inflammatory response, cell response to growth factor stimulation, actin reorganization, and regulation of cell proliferation (Fig. 4B). In contrast, genes showing an expression pattern similar to that of miR-200s, were primarily involved in ncRNA metabolism and RNA splicing, with a minor relevance of Wnt signaling pathway. We also observed a significant representation of chromatin remodeling and histone modification related genes in this category (Fig. 4B).

The analysis of possible oncogenic pathways involved (by overlap with MSigDB_Oncogenic_Signatures database) indicated that those genes following an expression pattern similar to that of miR-200 family are overexpressed upon *IL15* stimulation, *E2F1* or *MYC* overexpression, PRC2 or *BMI1* knockdown, βcatenin activation or *TP53* mutation, while they were downregulated upon *E2F3A* overexpression. A similar analysis of those genes displaying an inverse expression pattern, revealed a significant overlap with genes downregulated upon *E2F1* or *ATF2* overexpression, *TP53* mutation, or *AKT1* activation, while they are overexpressed upon *BMI1*, PRC2 or *PTEN* knockdown and *KRAS* or *LEF1* overexpression (Supplementary Tables 4 and 5).

Finally, we also used Chip Enrichment analysis [28] to find the putative binding of transcription factors to genes displaying an expression pattern similar or opposite to that of miR-200 family. This revealed that genes with an inverse pattern displayed binding sites to *SUZ12, STAT3, AR, PAX3, TP53, PPARG, EZH2, CTNNB1, EED, and BMI1*, whereas the genes following a similar trend to miR-200 display binding sites to *MYC, E2F1* and *E2F4* (Fig. 4C).

Collectively, these findings suggested that miR-200 family upregulation may have oncogenic consequences in BC. Moreover, our data also indicate the possible involvement of various oncogenic pathways, such as *TP53* alterations, E2F overexpression and *MYC* activation. In addition, these observations suggested that chromatin remodeling and histone modification processes occur in parallel with the increased expression of miR-200 elements.

### Down regulation of miR200 family is associated with poor prognosis in BC

**Figure 5:**
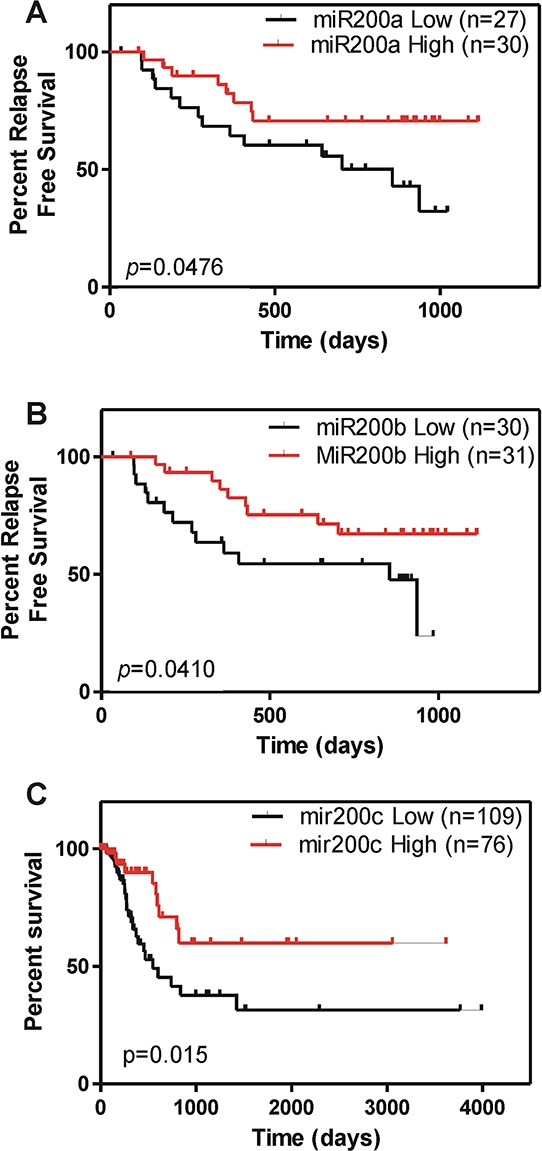
miR200 downregulation associates with poor clinical outcome in BC **A. B.** Kaplan Meyer analyses showing reduced expression of miR-200a (A) and miR-200b (B) associate with earlier recurrence in NMIBC tumors. **C.** Kaplan-Meyer analyses showing reduced overall survival in patients displaying low miR-200c expression from the external TCGA dataset.

The reduced expression of miR200 family members in T2 stage and high grade tumors (Fig. 1B and C) might indicate a potential role of such downregulation in tumors prone to recurrence. This aspect was further supported by Gene set enrichment analysis focused on miRNAs and aimed to discriminate recurrent and non-recurrent tumors. This revealed a primary involvement of CAGTATT, MIR-200B, MIR-200C, MIR-429 target gene set in recurrent tumors (NES = −1.69. *p* value < 0.0001; FDR *q* value = 0.029). To study whether the expression of the mir-200 family was associated with recurrence, we performed Kaplan Meyer analyses in our patient series. This showed that low levels of miR-200a and miR-200b were associated with increased likelihood of early tumor recurrence (Fig. 5A, B). This finding was reinforced by receiver operating characteristic curve (ROC) analysis (not shown), which indicated that reduced expression of miR-200a, miR-200b and miR-200c may provide an indicator for the detection of early recurrence (AUC for miR-200c = 0.728, *p* = 0.005; AUC for miR-200b = 0.711, *p* = 0.008; AUC for miR-200a = 0.704, *p* = 0.011). Similarly, using the TCGA data, we observed that those patients with lower expression of miR-200c showed a worse prognosis with reduced overall survival probability (Fig. 5C).

### Role of miR-200 and EMT in BC

The miR-200 family members are suppressors of the EMT program through the downregulation of various transcription factors [29–31]. In agreement, we also observed that genes showing an inverse expression pattern to miR-200 family are involved in cell migration (Fig. 4B). Consequently, we next studied the correlation between miR-200 family members and EMT modulators. No significant correlation was found between miR-200 family and *ZEB1* or *ZEB2* in NMIBC samples (Supplementary Figure 1A, 1B). Similar findings were obtained when other transcription factors controlling the EMT process (*SNAIL, SLUG, TWIST*) were also studied (Supplementary Figure 2A, 2B, 2C). Furthermore, no association was observed between the expression levels of these transcription factors and the clinicopathologic characteristics and/or outcome of NMIBC patients (not shown). These results suggested that the EMT-promoting transcription factors are not the primary targets of the miR-200 family in NMIBC, and indicate that, in these tumors, the EMT process is not directly related to increased recurrence.

**Figure 6:**
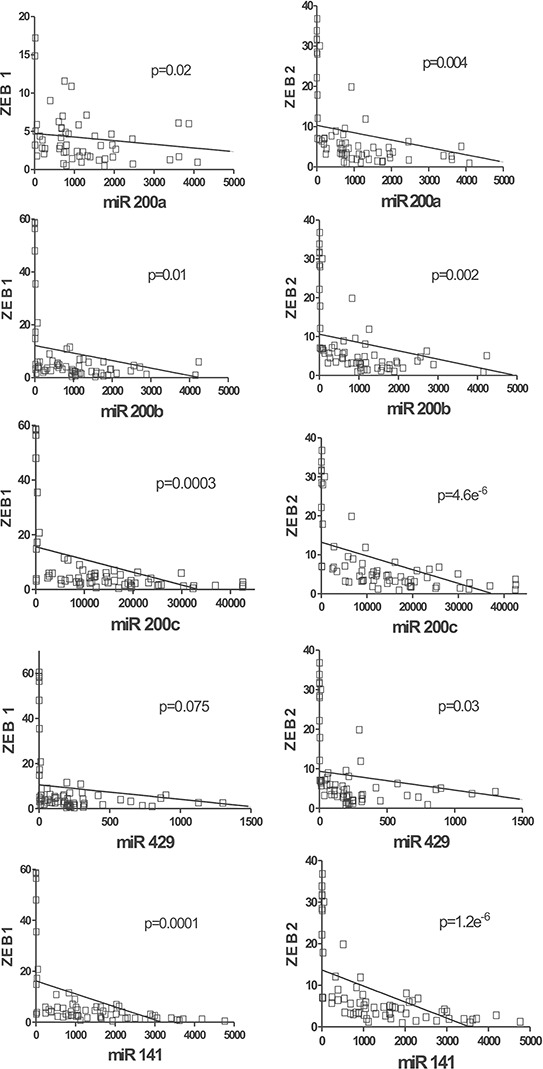
miR-200 expression negatively correlates with *ZEB1* and *ZEB2* in MIBC RNA seq data corresponding to *ZEB1* **A.** or *ZEB2* **B.** genes were represented as a function of the corresponding miR-200 family member using the data of MIBC samples present in the TCGA external dataset. *P* values of the Pearson correlation between the members of miR-200 family and *ZEB1* and *ZEB2* are provided.

This unexpected observation prompted us to analyze whether this absence of correlation between miR-200 family and *ZEB1/2* could be also extensive to MIBC. Using the corresponding RNA-seq data from the TCGA database, we observed a clear inverse correlation between the miR-200 family expression (especially for the cluster 2) and their targets *ZEB1* and *ZEB2* (Fig. 6). These results indicate that miR-200 family members could regulate the EMT processes in MIBC through their action on *ZEB1* and *ZEB2*. However, we did not detect any significant association between the expression of these EMT-regulating transcription factors and the clinical outcome of MIBC patients included in the TCGA database (Supplementary Figure 3).

### MiR-200 family and BMI1 in NMIBC

**Figure 7:**
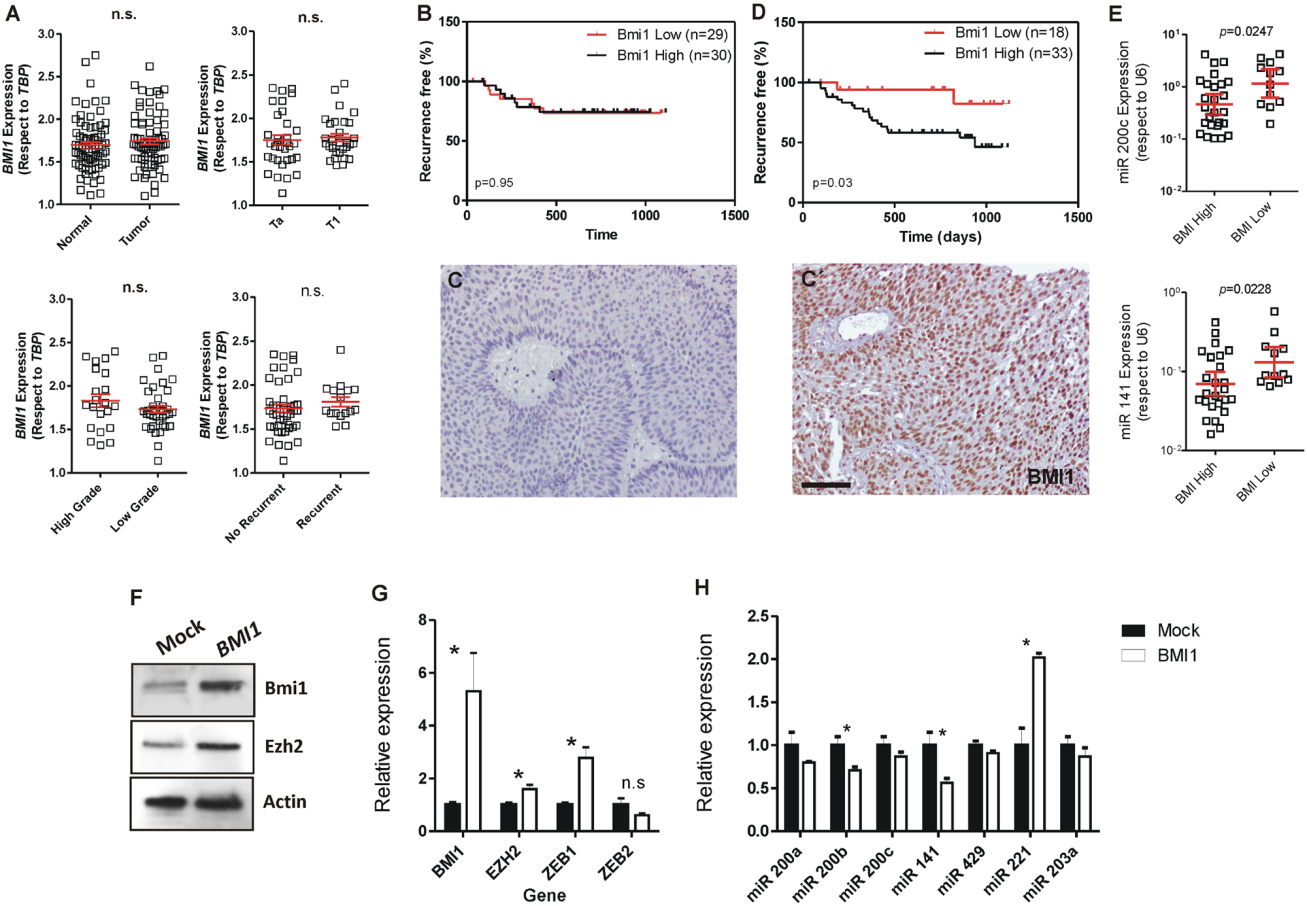
BMI1 protein levels associate with early recurrence in NMIBC and negatively correlates with miR-200 family expression **A.** *BMI1* gene expression measured by qPCR showing no significant differences between normal and tumor samples, or between stages, grades or recurrence onset in NMIBC dataset. **B.** Kaplan-Meyer analysis of NMIBC recurrence using *BMI1* gene expression (according the median) in NMIBC showing no significant differences. C, C') Examples of negative **C.** and positive (C') BMI1 staining in the tissue microarray of NMIBC samples. **D.** Kaplan-Meyer analysis of NMIBC recurrence using BMI1 protein expression showing that positive staining is associated with earlier recurrence. **E.** The high expression of BMI1 protein associates with reduced expression of miR-200 family members belonging to the cluster 2 (miR-200c and miR-141). **F.** Immunoblot showing the expression of BMI1 and EZH2 in RT112 bladder cancer cells upon transfection with human *BMI1* gene compared to control (Mock) transfected cells. **G.** Expression of *BMI1, EZH2, ZEB1 and ZEB2* genes as measured by qPCR in *BMI1*-transfected RT112 cells with respect to control (Mock) cells. H) Expression of the quoted miRNAs as measured by qPCR in BMI1-transfected RT112 cells with respect to control (Mock) cells. Bar in C' = 150 μm.

*BMI1* is a reported miR-200 target gene [32], and increased BMI1 expression has been associated with poor prognosis in bladder cancer patients [20, 33]. Furthermore, our microarray analysis also suggested an inverse relationship between the upregulation of miR-200 family and BMI1-mediated changes in gene expression (Supplementary Tables 4 and 5). We thus analyzed *BMI1* gene expression in our patient series. This revealed no significant differences between normal and tumor samples, stage, grade or recurrence (Fig. 7A). In addition, *BMI1* mRNA levels could not discriminate patient series according recurrence-specific survival (Fig. 7B). However, increased BMI1 protein levels (as assessed by immunohistochemistry in tissue microarrays (Fig. 7C, C') were associated with early recurrence (Fig. 7D).

This discrepancy between gene and protein expression in predicting clinical outcome might suggest a possible posttranscriptional regulation of *BMI1* gene. When we determined the BMI1 protein levels according the relative expression of miR-200 family members, we observed that the cluster 2 of miR-200 showed a negative correlation with the protein levels of BMI1, in agreement with the mechanism previously proposed by Cao et al. [32] (Fig. 7E).

We next analyzed whether BMI1 could also regulate miR-200 expression. To this, we transiently overexpressed human BMI1 protein in RT112 bladder cancer cells (Fig. 7F). We found that this produced moderate increase in the expression of EZH2 and ZEB1 without significant changes in ZEB2 (Fig. 7F, 7G). Importantly, we also observed a significant reduction of miR200b and miR141 and the increase in miR221 expression levels upon increased expression of BMI1 (Fig. 7H). These results suggest that BMI1 is able to repress the expression of, at least, some miR200 family members.

### Role of EZH2 in miR200 downregulation

**Figure 8:**
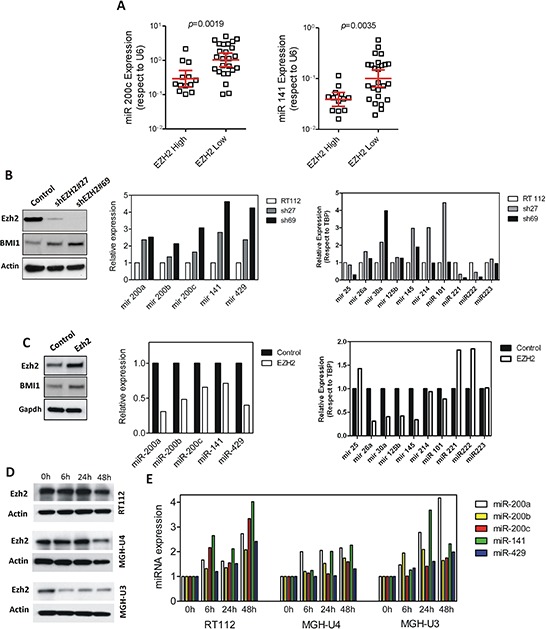
EZH2 regulates miR200 family members **A.** Distribution of miR-200 family members corresponding to the cluster 2 according to the high and low EZH2 protein levels determined by staining of tissue microarrays. **B.** Knockdown of EZH2 produces increased miR200 family expression in RT112 bladder cancer cells. Left panel: Western blot showing EZH2 and BMI1 protein expression in control and in two different silenced derivatives; center panel: qPCR analyses showing the miR-200 family expression in the corresponding cells; right panel: qPCR analyses showing the altered expression of various miRNAs previously associated with BC development or progression upon knock down of *>EZH2* gene. **C.** Increased expression of EZH2 induces reduction of the miR200 family members. Left panel: Western blot showing EZH2 and BMI1 expression in vector or in *EZH2*-transfected cells; center panel: qPCR analyses showing the miR200 family expression in the corresponding transfected cells; right panel: qPCR analyses showing the altered expression of various miRNAs previously associated with BC development or progression in transfected cells relative to mock-transfected cells. **D.** Western blot showing the expression of EZH2 in the quoted bladder cancer cells treated for the stated time periods with the EZH2-specific inhibitor DZNep (10 μM). Note that EZH2 levels were only significantly reduced in MGH U3 and in MGH U4 after 48 hours of DZNep treatment. **E.** qPCR showing the relative expression of the miR-200 family members after EZH2 inhibition mediated by incubation with DZNep (10 μM) for the quoted time periods in the RT112, MGHU4 and MGHU3 bladder cancer cell lines. Values were normalized according the expression of each miR200 member observed in the absence of DZNep treatment. Actin or GAPDH was used to normalize protein loading in Western blot analyses.

Besides methylation, aberrant miR-200 repression has been associated with transcriptional repression by EZH2 [32]. We have recently shown that increased EZH2 protein is a common hallmark of NMIBC at high risk of recurrence and tumor progression [21]. In agreement, our microarray data also indicated an inverse correlation between the expression of miR-200 family and those genes controlled by PRC2 (Fig. 4C and Supplementary Tables 4 and 5). Further, the forced expression of BMI1 caused a moderate increase of EZH2 expression and the decrease of miR-200 family members (Fig. 7F-H). To analyze the possible relationship between EZH2 and miR-200, we first characterized the expression of miR-200 members in our tumor series according the increased or reduced expression of EZH2 protein (also monitored by immunohistochemistry in tissue microarrays [21]). We found that BC samples characterized by increased EZH2 expression displayed reduced levels of miR-200c and miR-141 (Fig. 8A). In agreement, knockdown of EZH2 in RT112 bladder cancer cells produced the overall upregulation of all miR-200 members (Fig. 8B) without significant changes in BMI1 protein (Fig. 8B). Similarly, the overexpression of EZH2 caused the reduction in all miR200 family member expression (Fig. 8C). Indeed, we also found an inverse correlation of EZH2 (eg. decreased expression upon EZH2 knock down and increased expression upon EZH2 overexpression) and the expression of miR-221, which facilitates EMT in bladder cancer [34] and miR-222, which is associated with poor outcome in BC patients [35, 36] (Fig. 8B, 8C). In contrast, miR-30a and miR-145, which are suggested to act as tumor and/or EMT suppressors [37, 38], are upregulated by *EZH2* silencing, and are repressed upon EZH2 overexpression (Fig. 8B, 8C), in agreement with others [39]. These results indicate that the effects of EZH2 increased expression are not due to overall decreased miRNA expression. Finally, to monitor whether the EZH2 activity is required for miR-200 repression, we carried out a pharmacological EZH2 inhibition using DZNep in three different bladder cancer cell lines. We observed that incubation with DZNep at different time points, even when only a modest reduction in EZH2 protein expression was achieved (Fig. 8D), promoted a substantial increase in miR200 family expression (Fig. 8E). Collectively, these results indicated that EZH2 activity negatively controls the miR200 family expression in bladder cancer.

## DISCUSSION

Bladder cancer is a current challenge in medical practice as there is paucity in specific biomarkers that may predict the clinical outcome of the patients, or that may help in the development of new therapeutic approaches. The large scale integrated genomic analysis could help to identify novel biomarkers [40–42]. Among the possible biomarker candidates, miRNAs have gained intense attention.

The miR-200 family has been widely reported as potential metastasis suppressors in multiple tumor types. Deregulated expression of miR-200 family members has been reported in several types of tumors, including bladder cancer [43–45]. Recently, in addition to their well characterized role as inhibitors of the EMT program, their importance in the context of cancer stem cell self-renewal and differentiation, modulation of cell division and apoptosis, and resistance to chemo-resistance, has been also demonstrated [46]. Here, we show that the whole miR-200 family display higher expression in tumor compared to normal bladder samples, both in NMIBC and MIBC. This finding, which is in agreement with previous observations [43, 47] seems to be at odds with the widely reported activity of these miRNAs as negative regulators of the EMT/PRC/CSC [48, 49]. Here we provide evidences indicating that loss of methylation could be a possible molecular mechanism driving the overexpression miR-200 family. Whether this hypomethylation confers any oncogenic potential, or is just a consequence of global hypomethylation during bladder tumorigenesis remains undetermined and will be the subject of future research. It has been previously shown that methylation play also a role during cancer progression modulating the expression of both miR-200 clusters in other tumor types [50–54], and in MIBC cell lines [55]. Nonetheless, our microarray analyses strongly support that upregulation of the miR-200 family is mediated by the activation of specific oncogenic pathways, such as E2F or c-myc overexpression, whilst is also acting against others, such PRC2- and PRC1-induced gene expression changes. Our analysis, based on TCGA data, also showed that, methylation status of miR-200 family members seems to vary among tumor grades, being significantly higher in high-grade tumors, consistent with the reduced miR-200 expression.

Epithelial tumor cells undergo EMT for successful dissemination and, accordingly, we found a tendency of lower miR-200 expression in high grade and stage tumors in both NMIBC and MIBC. Our results are in agreement with Lee et al [56] who showed higher levels of miR-200c in low grade tumors, and proposed that these miRNAs could act as bladder tumor suppressor with gradually decreasing expression levels with tumor progression. In a similar context, Wiklund et al [55] also found lower levels in invasive compared with superficial tumors. Importantly, when looking at the role of miR-200 in disease outcome, we observed a similar trend for NMIBC and MIBC, as the lower the expression, the worse is the prognosis according to recurrence (NMIBC) or to overall survival (MIBC). Therefore our observations support the hypothesis that the reduced miR-200 expression could be used as a potential biomarker for poor bladder cancer outcome. Remarkably, the recently reported tumor suppressor activity of Notch in bladder tumorigenesis [57] is related to the EMT repression, at least in squamous-like BC subtype, which also associated with poor clinical outcomes [58]. The possibility that Notch may also act through the modulation of miR-200 family is an attractive possibility to be analyzed in future experiments.

The role of miR-200 family in EMT has been associated to its capacity to inhibit the expression of *ZEB1* and *ZEB2* in multiple human tumors [29–31]. ZEB1/2 facilitate EMT by efficiently inhibiting the expression of the cell–cell adhesion molecule E-Cadherin. In MIBC tumor samples, we observed reduced levels for both *ZEB1* and *ZEB2*, and a negative correlation between the expression of the miR-200 family and both *ZEB* genes. Therefore, these data may support an involvement of EMT in MIBC progression. However, in the case of NMIBC, no association was found between the expression of miR200 family members and the transcription factors mediating EMT, suggesting that these transcription factors are not primary targets of miR-200 in NMIBC. Currently, the actual role of EMT in BC outcome is still inconclusive due to the variability of results found in literature [59–63], and a possible different role of EMT in MIBC and NMIBC has been proposed [60, 61, 63].

The PRCs also participate in promoting EMT and conferring stem cell phenotypes [16, 49]. BMI1 (a member of the PRC1) has recently been shown to be involved in tumor progression and metastasis, probably mediating CSC function [49]. Our data show that increased BMI1 protein expression associates with earlier recurrence. Nonetheless, this relationship is only found when protein levels, but not gene levels, were analyzed, suggesting that BMI1 is posttranscriptionally regulated in BC. In agreement, we observed increased BMI1 protein levels in samples showing reduced miR-200 family expression. These results indicate that BMI1 is a potential target of miR200 family members, in agreement with other tumor types [32]. Importantly, our data show reduced expression of miR-200 members upon BMI1 overexpression, thus suggesting the existence of a possible feedback loop between BMI1 and miR-200 expression. Regarding PRC2, we have recently demonstrated that elevated EZH2 expression promotes extensive gene expression rewiring leading to increased tumor recurrence and progression in human NMIBC. Our results, using bladder cancer cell lines, extend this observation by showing that EZH2 expression may also lead to miR-200 family repression. Accordingly, tumors showing increased EZH2 expression also display reduced miR-200 expression leading to early recurrence. Remarkably, our pharmacological and knockdown experiments may provide a future therapeutic strategy. The inhibition of the EZH2 expression or activity not only would prevent the changes in gene expression leading to tumor recurrence, but also could preclude tumor progression. In addition, the roles of EZH2 do not seem to be limited to miR-200 expression, as other various miRNAs with potential oncogenic (miR-221, miR-222 [34–36]) or tumor suppressor (miR-30a, miR-145 [37, 38]) activity in bladder also appear to be modulated by EZH2. The availability of genetically engineered mice recapitulating the EZH2 oncogenic activity in bladder cancer [21] warrant preclinical test of this possibility.

**Figure 9:**
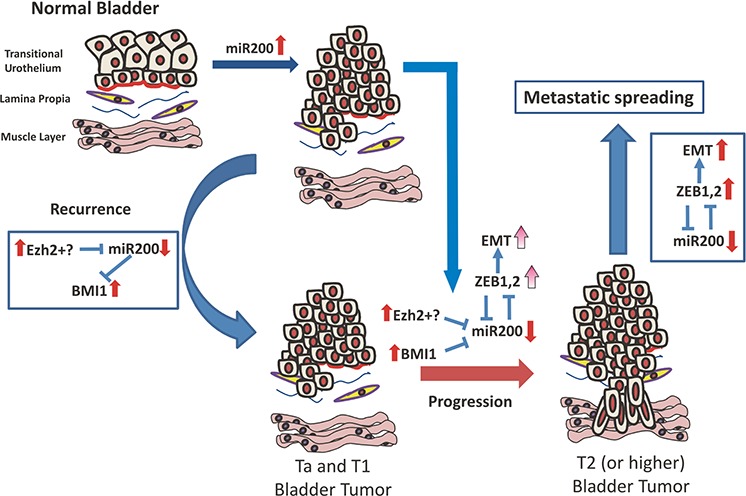
Possible mechanism integrating Polycomb and miR200 deregulated expression during bladder cancer progression and recurrence (see text for explanation)

In summary, our present data support a possible model of bladder cancer progression through an epigenetic mechanism of coordinated deregulation of the PRC1 and PRC2, and the miR-200 family (Fig. 9). Accordingly, tumors arising in the urothelium display an increased expression of miR-200 family members, in part mediated by hypomethylation and probably mediated by specific oncogenic challenges. Under this context, increased BMI1 and/or EZH2 expression contributes to miR-200 downregulation. This allows the increased BMI1 expression, which together with EZH2 facilitates NMIBC recurrence. Furthermore, increased expression of EZH2 and BMI1 results in additional downregulation of miR-200 members, which leads to the subsequent upregulation of EMT-promoting transcription factors and favors the invasive behavior of the tumor cells and the progression into MIBC. In view of the distinct molecular mechanisms acting in NMIBC and MIBC, the possible association of miR200 expression patterns with different mutational and genomic alterations is an attractive future research goal.

## MATERIALS AND METHODS

### Patients

Tumor samples and medical records were analyzed from 87 patients (pathologic and clinical data are given in Supplementary Table 1) who had been consecutively evaluated at the Urology Department of the University Hospital “12 de Octubre” between January 2009 and December 2012 [64]. Tumor samples were collected by multiple cold-cup biopsies from the exophytic part and from normal mucosa of the bladder of patients undergoing transurethral resection. All samples were kept in RNAlater. Several tumor and distant mucosa samples were fixed in formalin for conventional pathology diagnoses. The histopathology status was confirmed by the Pathology Department of the University Hospital “12 de Octubre” following latest WHO and TNM guidelines. The *FGFR3* and *PIK3CA* gene status of the tumors has been previously reported [64]. All patients were followed within a local program according to EAU guidelines. Informed consent was obtained from all patients and the study was approved by the Ethical Committee for Clinical Research of University Hospital “12 de Octubre”.

### RT-qPCR

Total RNA was isolated using miRNeasy Mini Kit (Qiagen) according to the manufacturer's instructions and DNA was eliminated (Rnase-Free Dnase Set Qiagen). Reverse transcription was performed for 10 ng total RNA using the Omniscript RT Kit (Qiagen) and specific primers for each gene. The sequences of the oligonucleotides used are listed in Supplementary Table 6. PCR was performed in a 7500 Fast Real Time PCR System using Go Taq PCR master mix (Promega) and 1 μl of cDNA template. Melting curves were performed to verify specificity and absence of primer dimers. Reaction efficiency was calculated for each primer combination, and TBP gene was used as reference gene for normalization [65]. To measure miRNA expression quantitatively, RNA was extracted using the same method as for the genes. Reverse transcription was carried out from 10 ng total RNA along with miR-specific primer using the TaqMan^®^ MicroRNA Reverse Transcription Kit (Applied Biosystems). PCR assays were performed using TaqMan^®^ Gene Expression Master Mix and 7500 Fast Real Time PCR System (Applied Biosystems). For normalization, we used RNU6B. Discrimination between samples showing increased or decreased tumor/normal relative expression was calculated using the median.

### Microarray analysis

To determine miRNA differential expression between normal and tumor bladder cancer samples we used LIMMA approach in a recently reported dataset performed in the Affymetrix HuGene-1_0-st-v1 platform [21] and deposited in GEO number (GSE38264). To detect genes with similar or opposite expression pattern with respect to miR200s, we used the Plavidis Template Matching adjusted to absolute R, *p* ≤ 0.005 in TMEV web utility. Unsupervised hierarchical clustering was performed using Pearson correlation and average linkage. Gene Ontology, mSigOncogenic signatures, mSig miRNA signatures and Chip Enrichment Analysis were performed using ChEAEnrich tool [28] as previously reported [66].

### Tissue Microarrays (TMA) and Immunohistochemistry

To determine BMI1 protein expression, we carried out immunohistochemistry analyses using an anti BMI1 antibody (Abnova MAB10506) and an anti EZH2 antibody (Abnova MAB9542), as previously reported [21, 64]. Signal was amplified using avidin-peroxidase (ABC elite kit Vector) and visualized using diaminobenzidine as a substrate (DAB kit Vector). Negative control slides were obtained by replacing primary antibodies with PBS (data not shown). Scoring of the results and selection of the thresholds, internal controls for reactivity of each antibody, and tissue controls for the series were done by double blind method according previously published methods [64]. At least two representative duplicate cores for each case were scored.

### Cell culture and transfection

Bladder cancer RT112, MGH U3 and MGH U4 cells were cultured in DMEM containing 10% FBS. Transfection experiments to upregulate EZH2 expression were performed using FuGENE^®^6 Transfection Reagent (Promega) and a plasmid coding for the cDNA of human EZH2 (from pGEX-EZH2, Addgene, USA) amplified by conventional PCR with the primers EZH2-for5′..GCCGAGCTAGCATGGGCCAGACTGGGA..3′ and EZH2-rev5′..GCCGAGGTACCTCAAGGGATTTCCATT TCTC..3′ and cloned into pCDNA 3.1 + (Hygro) mammalian expression vector (Invitrogen) under CMV promoter. The selection of the transfected cells was performed for at least 15 days in hygromycin (250 μg/ml; Sigma Aldrich) containing medium and pooled clones were used. For the knockdown of EZH2, the same cell line was transfected with 2 independent lentivirus-based shRNAs (MISSION^®^ shRNA, Sigma Aldrich) targeting human EZH2 gene (TRCN0000353069, denoted as sh27, and TRCN0000286227, denoted as sh69). Cells were selected by puromycin (0.5 μg/ml; Sigma Aldrich) resistance for 2 weeks and pooled clones were collected. For the cloning of human *BMI1* gene, total RNA was extracted from RT4 bladder cell line and 1 μg was reverse transcribed using the Omniscript RT Kit (Qiagen). Specific PCR amplification was performed by conventional PCR with BMI-specific primers 5′ GCGGATCCATGCATCGAACAACGAGAATCAAG 3′ (forward) and 5′GCCTCGAGTCAACCAGAAGAA GTTGCTGATGAC 3′ (reverse) and cloned into the BamHI and XhoI restriction sites of pCDNA 3.1 + (Hygro) mammalian expression vector (Invitrogen) under CMV promoter. Transfection experiments to upregulate BMI1 expression were performed using FuGENE^®^6 Transfection Reagent (Promega). The transfected cells were collected after 48 hours post-transfection and analyzed by qRT-PCR and/or immunoblot. Control, empty vector transfected cells (mock) were also generated in parallel. Pharmacological EZH2 inhibition was performed incubating cultures for 6, 24 and 48 hours in the presence of DZNep 10 μM.

### Western blot

Western blot was performed as described previously [21]. Briefly, pelleted bladder tumor cells were disrupted by freeze-thawing cycles in lysis buffer (200 mM HEPES pH 7.9, 25% glycerol, 400 mM NaCl, 1 mM EDTA, 1 mM EGTA, 1 μg/mL aprotinin, 1 μg/mL leupeptin, 1 mM PMSF, 20 mM NaF, 1 mM NaPPi, 1 mM Na_3_VO_4_, 2.5 mM DTT), and centrifuged to obtain supernatant containing total protein. 35 μg protein per sample were resolved in SDS-PAGE gels and transferred to nitrocellulose membranes (Amersham). Membranes were blocked with 0.1% Tween-20 with 5% BSA diluted in TBS and incubated with the appropriate antibodies diluted in TBS-T 0.5% BSA. Super Signal West Pico Chemiluminscence Substrate (Pierce) was used according to the manufacturer's recommendations to visualize the bands. Antibodies used are anti EZH2 (Abnova MAB9542), anti BMI1 (Santa Cruz Biotechnology sc-10745), anti Actin antibody (Santa Cruz Biotechnology sc-1616) and anti GAPDH antibody (Santa Cruz sc-25778).

### Statistical analysis

Comparisons were performed using the Wilcoxon–Mann–Whitney test, Limma and Student's *t* Test for paired samples showing gaussian distribution. Correlations were calculated by Pearson's Correlation. Survival analyses (recurrence/survival free) according to various variables were performed using the Kaplan–Meyer method and differences between the patient groups were tested by the log-rank test. SPSS 17.0, R statistical software v2.15.1 and Graphprism 5.0 software were used.

## SUPPLEMENTARY FIGURES AND TABLES


