# Supplementary material for: A Polycomb-mir200 loop regulates clinical outcome in bladder cancer
Source: Oncotarget. 2015 Oct 17;6(39):42258–75. doi: 10.18632/oncotarget.5546 (PMC4747223; doi:10.18632/oncotarget.5546)
Supplement: Supplementary file 1 [file oncotarget-06-42258-s001.pdf]

SUPPLEMENTARY TABLES AND FIGURES

Supplementary Table S1: Baseline Characteristics of the patients and Clinicopathological results in the series

**Patients (*n*)**	**87**
Age median (range)	73.0 yr (49–90)
Sex	M = 68<br>F = 19
Smoker status	No = 15<br>Currently smoker = 27<br>Ex smoker = 43<br>ND = 2
Stage	Papilloma = 1<br>Ta = 40<br>T1 = 36<br>T2 = 10
Grade	Low = 45<br>High = 37<br>PUNLMP = 4<br>ND = 1
Alterations in normal mucosa	Dysplasia = 6<br>Metaplasia = 1<br>Glandular cystitis = 1
Follow up, median (range)	864 d (34–1123)
Tumor size, median (range)	2 cm (0.5–7)
Number of implants, median (range)	1(1–10)
Specific death events	7*
Non-specific deaths	4**
Recurrence events	ND = 11<br>No recurrence = 43<br>Recurrence = 33
Time to recurrence, median (range)	186 d (96–433)
Stage of recurrence	Ta = 12<br>T1 = 12<br>T2 = 3<br>ND = 6
Grade of recurrence	Low = 12<br>High = 12<br>PUNLMP = 1<br>CIS = 2<br>ND = 6

(*Continued*)

**Patients (*n*)**	**87**
Previous tumor (non bladder)	16
Treatment by local instillation	ND = 23<br>Yes = 15 (14 BCG; 1 mitomycin)<br>No = 52

ND= Not determined
* All deaths due to progression of invasive bladder cancer.
** One patient died due to a lymphoma, another of sepsis secondary to mycosis fungoides, another due to progression of gastric tumor and another of chronic obstructive pulmonary disease decompensation
PUNLMP: Papillary urothelial neoplasia of low malignant potential
CIS: Carcinoma *in situ*

Supplementary Table S2:
See Supplementary Table S2 file

Supplementary Table S3:
See Supplementary Table S3 file

Supplementary Table S4: Significant overlap of genes following the same expression pattern of miR-200 with oncogenic pathways from MSigDB_Oncogenic_Signatures database

Term	Description	Overlap	*P*-value	Adjusted *P*-value
E2F1_UP.V1_DN	Genes down-regulated in mouse fibroblasts over-expressing E2F1	45/193	5.4151E-08	3.8332E-06
ATF2_S_UP.V1_DN	Genes down-regulated in myometrial cells over-expressing a shortened splice form of ATF2 [Gene*ID* = 1386] gene.	44/187	6.0844E-08	3.8332E-06
P53_DN.V1_DN	Genes down-regulated in NCI-60 panel of cell lines with mutated TP53 [Gene*ID* = 7157].	44/192	1.1614E-07	5.4876E-06
BMI1_DN.V1_UP	Genes up-regulated in DAOY cells (medulloblastoma) upon knockdown of BMI1 [Gene*ID* = 648] gene by RNAi.	37/147	1.6521E-07	6.2451E-06
BMI1_DN_MEL18_DN.V1_UP	Genes up-regulated in DAOY cells (medulloblastoma) upon knockdown of BMI1 and PCGF2 [Gene*ID* = 648] [Gene*ID* = 7703] genes by RNAi.	36/145	3.2163E-07	1.0131E-05
KRAS.KIDNEY_UP.V1_UP	Genes up-regulated in epithelial kidney cancer cell lines over-expressing an oncogenic form of KRAS [Gene*ID* = 3845] gene.	35/145	8.2067E-07	2.201E-05
LEF1_UP.V1_UP	Genes up-regulated in DLD1 cells (colon carcinoma) over-expressing LEF1 [Gene*ID* = 51176].	42/195	9.3165E-07	2.201E-05
ATF2_UP.V1_DN	Genes down-regulated in myometrial cells over-expressing ATF2 [Gene*ID* = 1386] gene.	40/187	1.9556E-06	4.1068E-05
MEL18_DN.V1_UP	Genes up-regulated in DAOY cells (medulloblastoma) upon knockdown of PCGF2 [Gene*ID* = 7703] gene by RNAi.	33/141	2.986E-06	5.2722E-05
PTEN_DN.V1_UP	Genes up-regulated upon knockdown of PTEN [Gene*ID* = 5728] by RNAi.	40/191	3.0685E-06	5.2722E-05
AKT_UP.V1_DN	Genes down-regulated in mouse prostate by transgenic expression of human AKT1 gene [Gene*ID* = 207] vs controls.	39/187	4.4309E-06	6.4418E-05

Supplementary Table S5: Significant overlap of genes following the opposite expression pattern of miR-200 with oncogenic pathways from MSigDB_Oncogenic_Signatures database

Term	Description	Overlap	*P*-value	Adjusted *P*-value
IL15_UP.V1_UP	Genes up-regulated in Sez-4 cells (T lymphocyte) that were first starved of IL2 [GeneID = 3558] and then stimulated with IL15 [GeneID = 3600].	27/192	0.00492204	0.4552887
PRC2_EDD_UP.V1_UP	Genes up-regulated in TIG3 cells (fibroblasts) upon knockdown of EED [GeneID = 8726] gene.	28/193	0.00287295	0.4552887
E2F3_UP.V1_DN	Genes down-regulated in primary epithelial breast cancer cell culture over-expressing E2F3 [GeneID = 1871] gene.	25/183	0.0091101	0.48997298
MYC_UP.V1_UP	Genes up-regulated in primary epithelial breast cancer cell culture over-expressing MYC [GeneID = 4609] gene.	25/186	0.0108324	0.48997298
P53_DN.V1_UP	Genes up-regulated in NCI-60 panel of cell lines with mutated TP53 [GeneID = 7157].	25/194	0.01673918	0.51612458
BCAT_BILD_ET_AL_UP	Genes up-regulated in primary epithelial breast cancer cell culture over-expressing activated CTNNB1 [GeneID = 1499] gene.	8/49	0.05321878	0.99996626
PRC1_BMI_UP.V1_UP	Genes up-regulated in TIG3 cells (fibroblasts) upon knockdown of BMI1 [GeneID = 648] gene.	22/190	0.05862928	0.99996626
E2F1_UP.V1_UP	Genes up-regulated in mouse fibroblasts over-expressing E2F1 [GeneID = 1869] gene.	19/189	0.1817207	0.99996626

Supplementary Table S6: Primers used for qPCR

Name	Sequence
EZH2-RTspec	CCTGTCGACATGTTTTGGTC
EZH2-for	CGGTGTCAAACGCCAATAAA
EZH2-rev	CCGCTCCACTCCACATTCTC
VIM-RTspe	AAGATTGCAGGGTGTTTTCG
VIM-for	GACCAGCTAACCAACGACAAA
VIM -rev	GAAGCATCTCCTCCTGCAAT
CDH1-RTspec	TGCTTAACCCCTCACCTTGAAGG
CDH1-for	GGTCTGTCATGGAAGGTGCT
CDH1-rev	GATGGCGGCATTGTAGGT
ZEB 1-RTspec	GTGTAACTGCACAGGGAGCA
ZEB 1-for	TTTTTCCTGAGGCACCTGAA
ZEB 1-rev	TGAAAATGCATCTGGTGTTCC
ZEB 2-RTspec	TTCTTCTCGTGGCGGTACTT
ZEB 2-for	AGGAGCTGTCTCGCCTTG
ZEB 2-rev	GGCAAAAGCATCTGGAGTTC
SNAIL 1-RTspec	GGACAGAGTCCCAGATGAGC
SNAIL 1-for	GCTGCAGGACTCTAATCCAGA
SNAIL 1-rev	ATCTCCGGAGGTGGGATG
SLUG-RTspec	GTTGGTCAGCACAGGAGAAAATGCC
SLUG-for	TTCGGACCCACACATTACCT
SLUG-rev	TTGGAGCAGTTTTTGCACTG
TWIST-RTspec	CACGCCCTGTTTCTTTGAA
TWIST-for	GGGCCGGAGACCTAGATG
TWIST-rev	TTTCCAAGAAAATCTTTGGCATA
BMI 1-RTspec	ATGGTTGTGGCATCAATG AA
BMI 1-for	GCTTCAAGATGGCCGCTTG
BMI 1-rev	TTCTCGTTGTTCGATGCATTTC
TBP-RTspec	GTGTTTAAAATCTACATA
TBP-for	AGTGAAGAACAGTCCAGACTG
TBP-rev	CCAGGAAATAACTCTGGCTCAT

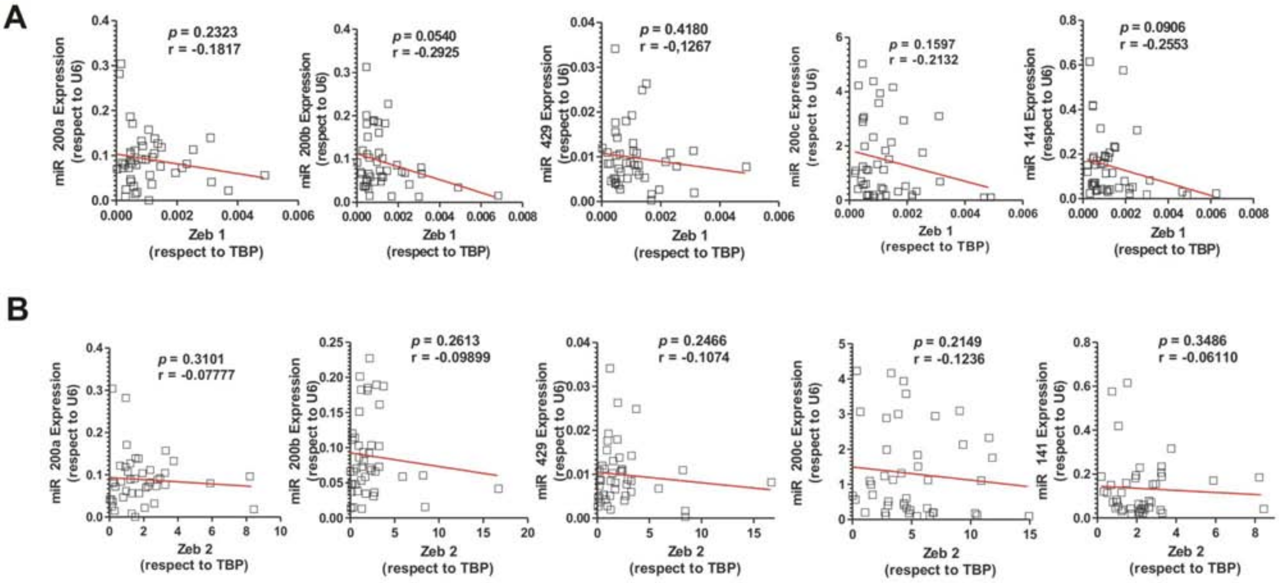

Supplementary Figure S1: Absence of significant correlation between the expression of miR-200 family members and ZEB1 A. and ZEB2 B. in NMIBC samples.

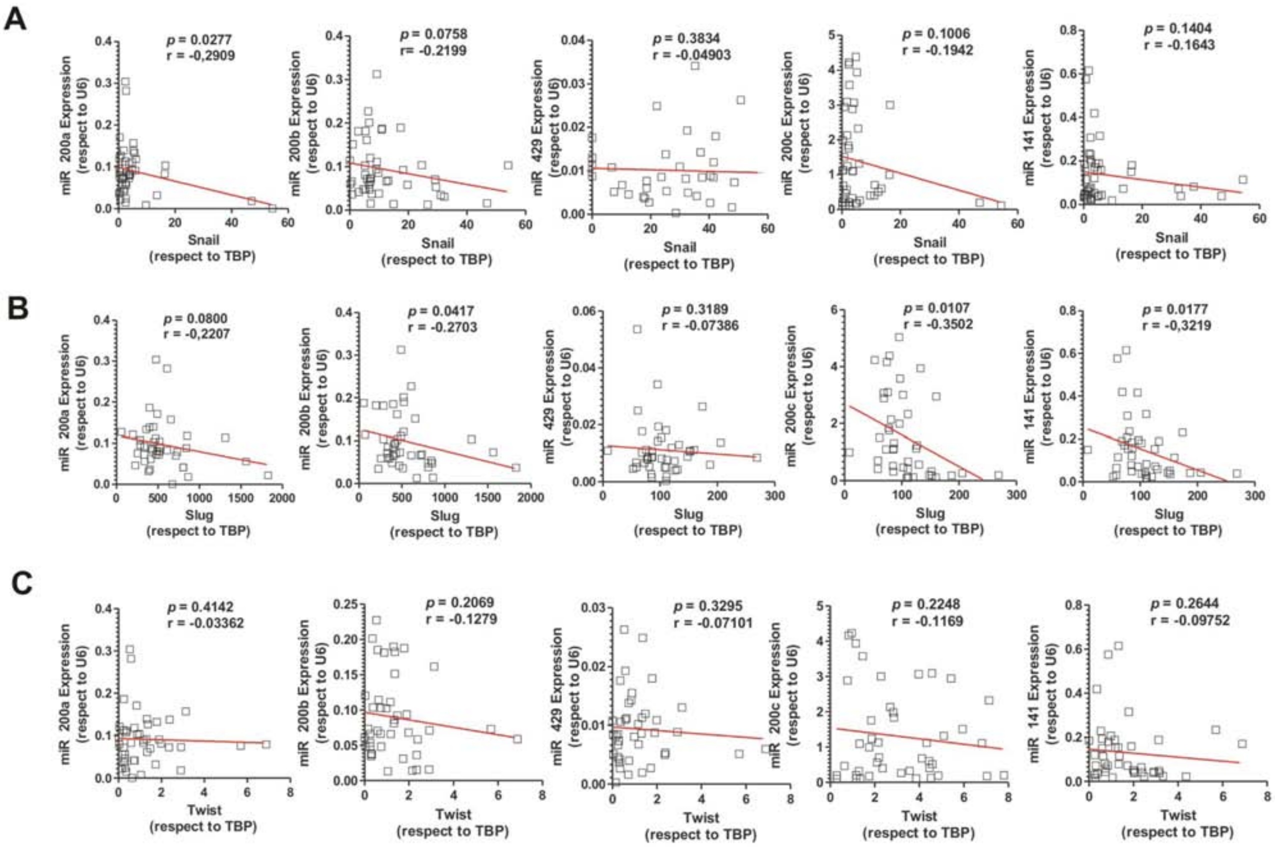

Supplementary Figure S2: Absence of significant correlation between the expression of miR-200 family members and *SNAIL* A. *SLUG* B. and *TWIST* C. in NMIBC samples.

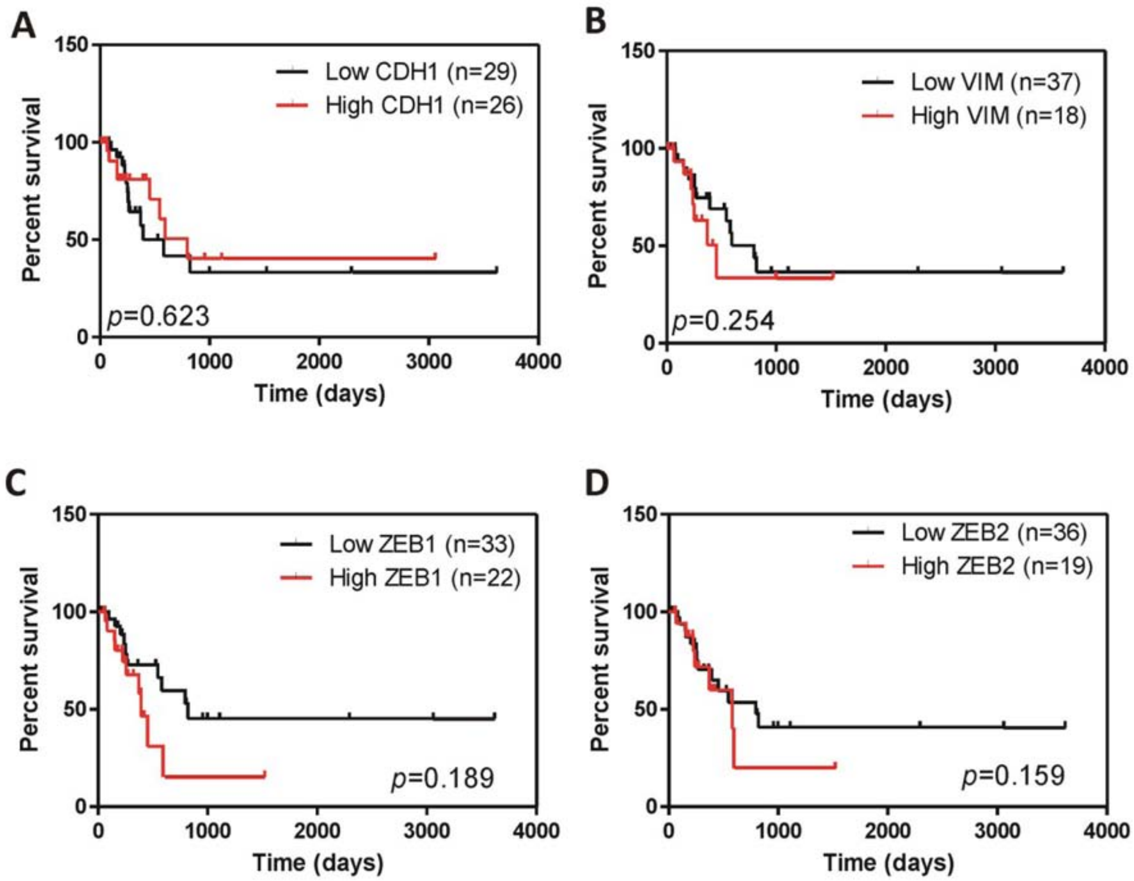

Supplementary Figure S3: Kaplan Meyer distribution corresponding to the overall survival patients included in the TCGA database according the expression of E cadherin A. Vimentin **B.** ZEB1 **C.** and ZEB2 **D.** Patients were discriminated by the median values of expression from RNA seq data. p Values were obtained by the log rank test.
